# KIF1A, R1457Q, and P1688L Mutations Induce Protein Abnormal Aggregation and Autophagy Impairment in iPSC-Derived Motor Neurons

**DOI:** 10.3390/biomedicines12081693

**Published:** 2024-07-30

**Authors:** Mingri Zhao, Junling Wang, Miao Liu, Yaoyao Xu, Jiali Huang, Yiti Zhang, Jianfeng He, Ao Gu, Mujun Liu, Xionghao Liu

**Affiliations:** 1 MOE Key Lab. of Rare Pediatric Diseases, Hunan Key Laboratory of Medical Genetics of the School of Life Sciences, Central South University, Changsha 410000, China; zhaomingri@sklmg.edu.cn (M.Z.); liumiao@sklmg.edu.cn (M.L.); 232511019@csu.edu.cn (Y.X.); huangjiali@sklmg.edu.cn (J.H.); zhangyiti@sklmg.edu.cn (Y.Z.); hejianfeng@sklmg.edu.cn (J.H.); guao@sklmg.edu.cn (A.G.); 2 Department of Neurology, Xiangya Hospital, Central South University, Changsha 410000, China; junling.wang@csu.edu.cn; 3 Department of Cell Biology, School of Life Sciences, Central South University, Changsha 410000, China; 4 Hunan Key Laboratory of Animal Model for Human Diseases, Central South University, Changsha 410000, China; 5 Hunan Key Laboratory of Basic and Applied Hematology, Central South University, Changsha 410000, China

**Keywords:** ALS, KIF1A, iPSC, motor neuron

## Abstract

Mutations in the C-terminal of KIF1A (Kinesin family member 1A) may lead to amyotrophic lateral sclerosis (ALS) through unknown mechanisms that are not yet understood. Using iPSC reprogramming technology and motor neuron differentiation techniques, we generated iPSCs from a healthy donor and two ALS patients with KIF1A mutations (R1457Q and P1688L) and differentiated them into spinal motor neurons (iPSC-MN) to investigate *KIF1A*-related ALS pathology. Our in vitro iPSC-iMN model faithfully recapitulated specific aspects of the disease, such as neurite fragmentation. Through this model, we observed that these mutations led to KIF1A aggregation at the proximal axon of motor neurons and abnormal accumulation of its transport cargo, LAMP1, resulting in autophagy dysfunction and cell death. RNAseq analysis also indicated that the functions of the extracellular matrix, structure, and cell adhesion were significantly disturbed. Notably, using rapamycin during motor neuron differentiation can effectively prevent motor neuron death.

## 1. Introduction

Amyotrophic lateral sclerosis (ALS) is an adult-onset neurodegenerative disease. The incidence of ALS is approximately 5 cases per 100,000 persons annually. It mainly affects motor neurons (MNs) in the brain stem and spinal cord. The death of motor neurons in the brain stem and spinal cord leads to gradual muscle weakness and atrophy in ALS patients, and patients ultimately die because of respiratory failure [1]. Nearly 10% of ALS cases are familial, and 90% are sporadic. To date, over 50 ALS-associated genes have been identified, including *SOD1*, *ANG*, *VCP*, *p62/SQSTM1*, *TDP-43*, *FUS*, *C9ORF72*, *KIF5A*, and *KIF1A* [2,3].

A variety of genetic, pathological, and neurobiological evidence suggests that deficits in axon transport play a crucial role in the pathogenesis of ALS [4]. Our previous research findings indicate that mutations in the C-terminal cargo-binding region of KIF1A may trigger the onset of ALS. Specifically, mutations at the C-terminus of KIF1A increase its binding affinity with transported cargoes such as RAB3 and VAMP2, highlighting the importance of KIF1A motor-mediated transport in ALS pathogenesis [5]. However, the mechanism by which KIF1A mutations at the C-terminal affect cargo binding remains unclear, and further research into its molecular mechanism is needed. Additionally, we did not observe neuronal death or morphological abnormalities in a previous mouse neuronal model, which we suspect is due to the presence of endogenous wild-type KIF1A. Studies have shown that overexpression of wild-type KIF1A can rescue neuronal morphological defects and prevent cell death caused by various mutations or truncated forms of KIF1A that interfere with normal cargo transport [6]. Overexpression of pathogenic KIF1A mutations in vitro may not fully mimic disease phenotypes and pathogenic mechanisms due to the presence of endogenous wild-type KIF1A. Therefore, it is essential to develop an endogenous neuronal model carrying pathogenic *KIF1A* mutations to elucidate how mutations in the C-terminus of KIF1A contribute to ALS.

Induced pluripotent stem cells (iPSCs) are a type of stem cells with the potential for unlimited proliferation and multidirectional differentiation [7,8,9,10]. In recent years, researchers have partially revealed the phenotype of ALS disease by reprogramming ALS patients’ somatic cells into induced pluripotent stem cells and guiding their differentiation into motor neuron cells (iMNs). To date, many findings have been obtained using the induced pluripotent stem cell-derived motor neuron (iPSC-MN) model, including the exploration of pathogenic mechanisms, drug screening, and the identification of novel therapeutic targets [11]. For example, many studies using the iPSC-MN model have revealed common neuropathological features, such as misfolded protein aggregates, axonal transport defects, axonal degeneration, and autophagy defects [12,13,14]. These findings demonstrate the effectiveness of the iPSC-MN model as a tool for exploring ALS pathophysiology.

In this study, we established an efficient method to reprogram peripheral blood T cells from voluntary donors into iPSCs. With the informed consent of the donors, we generated iPSCs from healthy volunteers and two ALS patients carrying heterozygous mutations in KIF1A (R1457Q, P1688L). The iPSC-MNs recapitulated the hallmark features of the human disease. We found that motor neurons carrying the KIF1A mutations R1457Q and P1688L exhibited morphological abnormalities such as neurite fragmentation and decreased adherence. RNA-seq analysis further revealed significant disturbances in the functions of the extracellular matrix, cellular architecture, and cell adhesion. In single-cell motor neurons, we observed that *KIF1A* mutations led to its aggregation at the proximal axon and abnormal accumulation of its transport cargo, resulting in autophagy dysfunction and cell death.

## 2. Materials and Methods

### 2.1. Isolation of Human T Cells and Generation of iPSCs

Peripheral blood mononuclear cells (PBMCs) were isolated from donors by centrifuging EDTA anticoagulant blood over a lymphocyte separation medium (His-topaque-1077, Sigma, Burlington, MA, USA), according to the manufacturer’s instructions. Subsequently, the T cells were obtained by using a Pan T Cell Isolation Kit (Miltenyi Biotec, Cologne, Germany). The cells were seeded on a 10 cm plate and cultured at 37 °C in 5% CO_2_ using ImmunoCult™-XF T cell expansion medium (Stem Cell Technologies, Vancouver, BC, Canada). After one day of culture, five million T cells were electroporated with pCE-hOCT3/4, pCE-hSK, pCE-hUL, pCE-mp53DD, and pCXB-EBNA1 (Addgene, Cambridge, MA, USA) using a Nucleofector 2D Device (Lonza), along with the Amaxa Human T cell Nucleofector Kit (Lonza). Ten hours after transfecting T cells with the plasmid, IL-2 (10 ng/mL) and an-ti-CD3/CD28 antibodies (25 µL/mL) were added to stimulate T cell proliferation. At 24 h post-electroporation, the medium was replaced with 1 mL of fresh N2B27 medium and 10 µM Y27632. The N2B27 medium comprised DMEM/F12, 1 × N-2 Supplement, 1 × B-27 Supplement, 0.1 mM MEM Non-essential Amino Acids Solution, 1 × GlutaMAX™-I, 100 µM β-Mercaptoethanol and 100 ng/mL bFGF (all from Thermo Fisher, Waltham, MA, USA). At 48 h post-electroporation, we carefully aspirated 1 mL of supernatant from the top of the well and then added 1 mL of N2B27 medium. We supplemented this daily, continuing this process for 5 days. On day 7, we carefully aspirated the spent medium and replaced it with 2 mL of N2B27 medium, continuing this process up to day 9 post-transfection. Next, we aspirated the spent N2B27 medium and replaced it with complete mTeSR Plus Medium (Stem Cell Technologies), continuing with daily changes. We expected the iPSC colonies to reach a suitable size for transfer within 20 to 30 days post-transfection.

### 2.2. Culture of iPSCs

The iPSCs were cultured in mTeSR Plus medium coated with Matrigel (Corning, NY, USA) in conditions of 5% CO_2_ at 37 °C. For passaging, when the cells reached 80% confluence, the iPSCs were digested with 0.5 mM EDTA for 5 min at room temperature. Then, the cells were subcultured at a ratio of 1:6 to 1:10. Finally, the harvested iPSCs were frozen using a solution of 10% DMSO (Sigma, Burlington, MA, USA) and 90% mTeSR Plus medium.

### 2.3. Teratoma Formation

The differentiation potential of the three germ layers of induced iPSCs was assessed by generating teratoma formation in vivo. This was achieved by injecting a 100 μL aliquot containing ten million cells, 50 μL DMEM/F12, and 50 μL Matrigel into the subcutaneous tissue near the axilla of nude mice. After 60 days, the teratoma was excised and fixed with 4% PFA for 24 h. It was then embedded in paraffin, sectioned, and stained with Hematoxylin and Eosin (HE) to evaluate its differentiation capacity into the three germ layers. All animal experiments were approved by the Institutional Animal Care and Use Committee of the Center for Medical Genetics at Central South University.

### 2.4. Chromosomal Karyotype

Cells at passage 20 were treated with colchicine (0.2 μg/mL) for 2 h at 37 °C. Subsequently, iPSCs were detached into single cells using TrypLE™ Select. The harvested cells were then incubated at room temperature with 0.075 M KCl for 15 min, followed by fixation in a 3:1 mixture of methanol and glacial acetic acid. Metaphase chromosome spreads were prepared by air-drying. The chromosomes were then heated at 75 °C and G-banded using Giemsa staining (Sigma).

### 2.5. Episomal Plasmids Detection

Genomic DNA from iPSCs at different passages was extracted using proteinase K digestion, followed by phenol and chloroform extraction, and finally isopropanol precipitation. PCR was then performed using the *EBNA-1* and *oriP* primer sets. The *EBNA-1* primer set is designed to detect all five episomal plasmids, while the *oriP* primer set can detect all episomal plasmids except for pCXBEBNA1, which lacks the *OriP* gene. To check for the absence of non-integrative episomal plasmids in iPSCs, the PCR products were subjected to 2% agarose gel electrophoresis. Primers used for PCR were the following: *EBNA-1*, forward: 5′-ATCGTCAAAGCTGCACACAG-3′; reverse: 5′-CCCAGGAGTCCCAGTAGTCA-3′; *oriP*, forward: 5′-TTCCACGAGGGTAGTGAACC-3′; reverse: 5′-TCGGGGGTGTTAGAGACAAC-3′ (Sangon Biotech, Shanghai, China).

### 2.6. Mutation Analysis

Genomic DNA was extracted and the PCR reaction was performed with specific primers of the *KIF1A* mutation. The mutation of the *KIF1A* gene was detected by Sanger sequencing at Tsingke Biotech (Beijing, China).

### 2.7. qRT-PCR Analysis

Total RNA was isolated from motor neurons using Trizol, and then 500 ng of extracted RNA was reverse transcribed into cDNA using HiScript II Reverse Transcriptase (Vazyme#R223, Nanjing, China), following the manufacturer’s instructions. Quantitative PCR was performed using the 2 × ChamQ Universal SYBR qPCR Master Mix (Vazyme#Q711). The *GAPDH* gene served as an endogenous control, and relative expression levels were calculated using the 2^−ΔΔCT^ method. Primers used for RT-PCR were the following: *CHAT*, forward: 5′-GGCCTTCTACAGGCTCCATC-3′; reverse: 5′-GTCAGTCACGGCTCTCACAA-3′; *FN1*, forward: 5′- CGGTGGCTGTCAG-TCAAAG-3′; reverse: 5′-AAACCTCGGCTTCCTCCATAA-3′; *COL1A1*, forward: 5′-GAGGGCCAAGACGAAGACATC-3′; reverse: 5′-CAGATCACGTCATCGCACAAC-3′; *COL1A2*, forward: 5′-GTTGCTGCTTGCAGTAACCTT-3′; reverse: 5′-AGGGCCAAGTCCAACTCCTT-3′; *GAPDH*, forward: 5′-GGCAAATTCCATGGCACCG-3′; reverse: 5′-GTTCACACCCATGACGAACA-3′ (Sangon Biotech, Shanghai, China).

### 2.8. RNA Sequencing Analysis

The mature motor neurons were collected after 25 days of culture for RNAseq. Briefly, the samples were sent to BGI Genomics for RNA sequencing analysis. The sequencing data were filtered with SOAPnuke (v1.5.2) software by removing reads containing sequencing adapters, reads with N content greater than 5%, and low-quality reads (BGI defines low-quality reads as those with bases having a quality score less than 15, where such bases constitute more than 20% of the total bases). Then, the clean reads were mapped to the reference genome (GRCh38.p13). We applied the DESeq2 algorithm to filter the differentially expressed genes. After the significance analysis, the *p*-value and FDR analysis were subjected to the following criteria: log2FC > 0.6 or <−0.6, *p*-value < 0.05, and FDR < 0.05. Gene Ontology (GO) analysis and KEGG pathway analysis were performed to elucidate the biological implications of the differentially expressed genes in the experiment. Fisher’s exact test was applied to identify significant GO categories (adjusted *p*-value < 0.05). For the KEGG pathway analysis, Fisher’s exact test was used to select significant pathways, with the threshold of significance defined as *p*-value < 0.05. Finally, we used the KEGG database to build the network of genes according to the relationships among the genes in the database.

### 2.9. Immunofluorescence

Cells were fixed with 4% paraformaldehyde (PFA) for 30 min. After three washes with DPBS, cells were permeabilized with 0.1% PBST (Dulbecco’s phosphate-buffered saline with 0.1% Triton X-100) for 30 min and then blocked with 5% bovine serum albumin in DPBS for another 30 min. Subsequently, cells were incubated with the primary antibody overnight at 4 °C. Following another three washes with DPBS, cells were incubated with various fluorescently labeled secondary anti-bodies and DAPI in blocking buffer for 1 h at room temperature. Images were acquired using a Leica LAS X SP-5 confocal microscope. Antibodies were purchased as indicated: OCT4 (Abcam, Cambridge, MA, USA), SOX2 (Abcam), NANOG (Abcam), SSEA4 (Abcam), TRA-1-60 (Abcam), OLIG2(Abcam), SOX1 (R&D Systems, Minneapolis, MN, USA), OTX2(R&D Systems), MNX1 (Sigma), SMI32 (BioLegend, Canada, USA), CHAT (Sigma), KIF1A (Abcam), Synaptophysin (Proteintech, Chicago, IL, USA). LAMP1 (Cell Signaling Technology, Danvers, MA, USA) and ATG9A (Proteintech, Tokyo, Japan).

### 2.10. iPSC Differentiation Into Motor Neurons

The differentiation of motor neurons derived from iPSCs was performed as previously described [15]. Briefly, iPSCs were dissociated with 1 mg/mL dispase and cultured in MN induction medium, including Neurobasal medium, DMEM/F12 at 1:1, 0.5 × N-2 Supplement, 0.5 × B-27 Supplement, 1 × GlutaMAX™-I (all from Thermo Fisher). Different combinations of CHIR-99021 (Selleck, Houston, TX, USA), DMH1 (Selleck), SB431542 (Selleck), Purmorphamine (Selleck), DAPT (Selleck), Retinoic acid (Sigma), Vitamin C (Sigma), and Valproic Acid sodium (Sigma) were added to the MN induction medium at different stages. When differentiating iPSCs into motor neuron progenitors, the cell clusters were dissociated into single cells using Accutase (Stem Cell Technologies), and then cultured in motor neuron (MN) induction medium containing 10 μM Y27632 for 48 h. Subsequent motor neuron induction followed the same procedure as described earlier.

### 2.11. CCK-8 Assay

The single motor neurons were cultured in 96-well plates at a density of 3000 cells per well in 100 μL of culture medium. Cell viability was measured on the first and sixth days using the Cell Counting Assay Kit (CCK-8, Vazyme) according to the manufacturer’s instructions. Briefly, CCK-8 was added to each well, and after 2–4 h, absorbance was measured at 450 nm using a microplate reader.

### 2.12. Western Blotting

Total protein lysates of cell samples were prepared using RIPA buffer (Thermo Fisher Scientific) in the presence of the protease inhibitor and PhosStop (Thermo Fisher Scientific). Protein concentrations were quantified with a BCA protein assay kit (Thermo Fisher Scientific). Proteins were denatured by heating at 100 °C for 10 min in Laemmli sample buffer containing β-mercaptoethanol. Equal amounts of protein were blotted onto PVDF membranes. The membranes were blocked for 2 h at room temperature in TBS containing 5% milk powder and 0.1% Tween-20, and subsequently immunoblotted with the corresponding primary antibodies overnight at 4 °C. After washing with TBST (TBS with 0.1% Tween-20), the membranes were incubated with horseradish peroxidase-conjugated secondary antibodies. Blots were visualized using the SuperSignal West Pico Substrate (Thermo Fisher Scientific) chemiluminescence kit and the Gene Genius Bioimaging System (Bio-Rad, Hercules, CA, USA). Antibodies were purchased as indicated: mTOR (Cell Signaling Technology), p-mTOR (Cell Signaling Technology), LAMP1 (Cell Signaling Technology), p62 (Cell Signaling Technology), LC3 (Cell Signaling Technology) and β-actin (Sigma).

### 2.13. Statistical Analysis

Statistical analyses were performed using GraphPad Prism 8.3 (GraphPad Software, Inc., San Diego, CA, USA, www.graphpad.com, accessed on 4 March 2021). Student’s *t*-test was used to compare the mean values of two groups. The data are expressed as mean ± SD. Statistically significant values were those lower than 0.05 and are indicated by an asterisk. * *p* < 0.05, ** *p* < 0.01, *** *p* < 0.001, and **** *p* < 0.0001.

## 3. Results

### 3.1. Generation of iPSC from Amyotrophic Lateral Sclerosis Patient with a Pathogenic KIF1A Mutation

**Figure 1:**
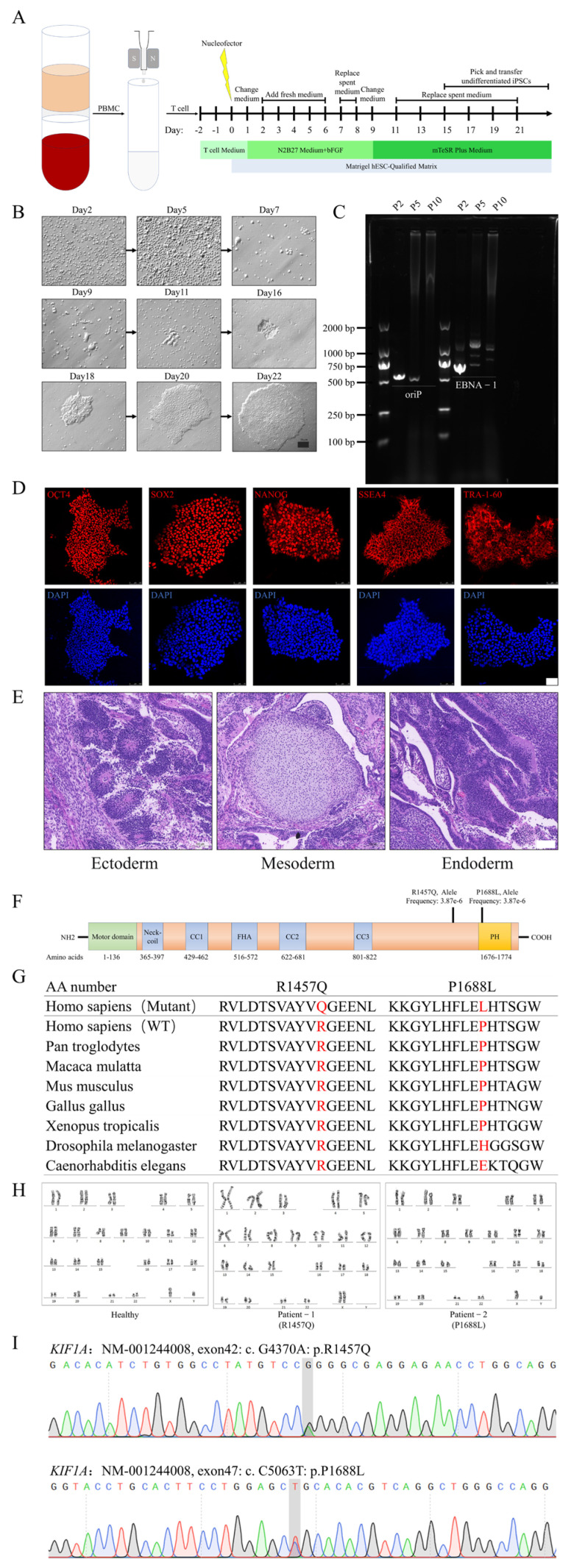
Generation of iPSCs from ALS patient with a pathogenic *KIF1A* mutation. (**A**) The iPSC induction protocol. (**B**) Morphological changes at different stages of iPSC induction from T cell. Scale bar: 50 μm. (**C**) Detecting foreign plasmids using PCR and agarose gel electrophoresis. (**D**) Immunofluorescence for early motor neuron markers MNX1 and SMI32 of iPSC expressing markers for OCT4, SOX2, NANOG, SSEA4, and TRA-1-60, the DAPI was used to visualize the nucleus. Scale bar: 75 μm. (**E**) In vivo teratoma assays with HE staining demonstrated that the iPSCs are capable of differentiating into all three germ layers: endoderm, mesoderm, and ectoderm. Scale bar: 100 μm. (**F**) A schematic showing the KIF1A protein and its mutations in ALS-related disorders. (**G**). Analysis of the evolutionary conservation of amino acids in the KIF1A protein that were altered in association with ALS. AA number, amino acid number of human KIF1A protein. (**H**) G-band karyotyping confirmed the diploid chromosome content in the iPSCs. (**I**) Sanger DNA sequencing of the iPSC line from the ALS patient revealed a mutation in the *KIF1A* gene. AA number, amino acid number of human KIF1A protein.

Our previous research identified *KIF1A* as a potential new pathogenic gene for ALS. With informed consent from healthy donors and ALS patients carrying *KIF1A* mutations, we established their iPSCs. The experiments were performed with blood from a healthy donor. Isolated peripheral blood T cells were reprogrammed by transducing the non-integrative *EBNA1/oriP* based episomal vectors, expressing the reprogramming factors OCT4, SOX2, LIN28, L-MYC, KIF4, and mp53DD. Ten hours after transfecting T cells with the plasmid, IL-2 and antibodies against CD3 and CD28 were added to stimulate the proliferation of T cells. Subsequent culturing was then performed in stages using N2B27 medium and mTeSR Plus medium (Figure 1A). At this early time point, these cells showed a round morphology and clear cell-to-cell boundaries. Thereafter, these cells gradually adhered to each other and formed tightly packed colonies. After 21 days, the iPSC clones were obtained (Figure 1B). The episomal plasmids detection shows that the iPSCs were free of foreign episomal plasmids after passage 10 (Figure 1C). We then characterized the iPSC colonies, and the immunofluorescence for early motor neuron markers MNX1 and SMI32 through staining of the iPSC was highly expressed in OCT4, SOX2, NANOG, SSEA4, and TRA-1-60 (Figure 1D). The teratoma formation in mice showed that iPSCs have the potential to differentiate into cells of all three germ layers (Figure 1E). In previous research, we found that the variants associated with the ALS phenotype were predominantly located in the C-terminal region of KIF1A (Figure 1F). Evolutionary conservation of altered amino acids of KIF1A mutation is associated with ALS, and we found that the amino acid at position 1457 of the KIF1A protein is highly conserved across multiple species, including Pan troglodytes, Macaca mulatta, Mus musculus, Drosophila melanogaster, and Caenorhabditis elegans, etc. (Figure 1G). Additionally, the amino acid at position 1688 in KIF1A is conserved among various mammals and in species such as Gallus gallus and Xenopus tropicalis. This conservation analysis suggests that mutations at these sites may have a higher likelihood of being pathogenic. For the successfully established iPSCs, after multiple passages and expansions, chromosomes from the iPSCs revealed a normal diploid content, as assessed by G-band karyotyping (Figure 1H). Sanger DNA sequencing of the iPSC revealed a heterozygous mutation c.4370G > A; p. Arg 1457 Gln and c.5063 C > T; p. Pro 1688 Leu in the *KIF1A* gene in iPSCs from the ALS patient (Figure 1I).

### 3.2. Abnormal Morphology of Motor Neurons with KIF1A Mutant

**Figure 2:**
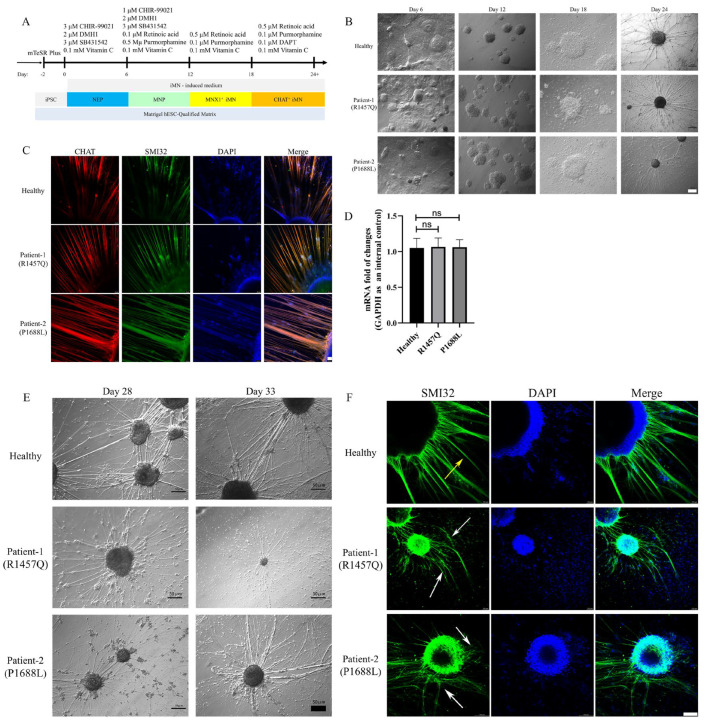
iPSC-derived motor neurons with the *KIF1A* mutation exhibit axonal morphology. (**A**) Schematic of the protocol for motor neuron differentiation. Abbreviations: NEP, neuroepithelial progenitor; MNP, motor neuron progenitor; iMN, induced motor neuron. (**B**) Bright-field images of motor neurons from a healthy donor and ALS patient iPSC differentiation. Scale bar: 50 μm. (**C**) Immunofluorescence for mature motor neuron markers CHAT and SMI32 on day 24. Scale bar: 50 μm. (**D**) *CHAT* gene expression in mature motor neurons from a healthy donor and ALS patient was quantified using qRT-PCR. (**E**) Bright-field images of motor neurons from a healthy donor and ALS patient on day 28 and day 33. Scale bar: 50 μm. (**F**) Immunofluorescence for mature motor neuron neurofilament by SMI32 on day 33. Normal: yellow arrow heads; Abnormal: white arrow heads. Scale bar: 100 μm. Data are shown as mean ± SD (ns, not significant; Student’s *t*-test was used for comparison).

Using a small-molecule cocktail, we differentiated iPSC lines from a healthy donor and ALS patient into spinal motor neurons (Figure 2A,B). By day 6, SOX1^+^/OTX2^+^ neuroepithelial progenitors (NEPs) were generated (Figure S1A,B). On day 12, OLIG2^+^ motor neuron progenitors (MNPs) appeared (Figure S1C,D), and by day 18, MNX1^+^ motor neurons were present (Figure S1E,F). By day 24 of differentiation, all iPSC lines had differentiated into CHAT+ spinal motor neurons, which serve as a marker for mature motor neurons (Figure 2C). To investigate the differentiation potential of iPSCs from a healthy donor and an ALS patient into motor neurons, we examined the morphology and proportion of neuron types during motor neuronal differentiation. In the early stages, motor neuron morphologies were the same for both healthy donors and ALS patients. Across each stage, motor neurons from both healthy donors and ALS patients showed no difference in the ratios of SOX1^+^, OTX2^+^, OLIG2^+^, and MNX1^+^ positive neurons (Figure S1). Through quantitative real-time PCR analysis, we assessed CHAT expression in mature motor neurons from both healthy donors and ALS patients. The results indicated no significant difference in CHAT expression between spinal motor neurons from healthy donors and ALS patients (Figure 2D). Overall, iPSCs from ALS patients carrying the KIF1A mutation can still differentiate into motor neurons, and the differentiation efficiency was not affected by the KIF1A mutant. However, ALS-related motor neuron pathology typically manifests post-maturation [15]. Thus, after inducing iPSCs to differentiate into mature motor neurons, we continued to maintain the culture and observed changes in neuronal cell morphology during this period. We found that as the culture period extended, motor neuron cell clusters from ALS patients began to float, with cells gradually dying and neurofilaments breaking. Axons of WT (healthy donor) MNs appeared smooth, thick, and firm (indicated as “normal”, as shown by yellow arrow head). In contrast, KIF1A mutant MNs exhibited damaged morphologies of fragile axons (SMI32, which marks axons by targeting non-phosphorylated neurofilaments) with more slender branchings (indicated as “abnormal”, as shown by white arrowheads) (Figure 2E,F). Motor neuron abnormalities are the most critical pathological phenotype of ALS. Therefore, we confirmed that ALS iPSC-derived motor neurons carrying the *KIF1A* mutation could recapitulate the disease-related phenotype in vitro.

### 3.3. RNA-Seq Analysis Shows That KIF1A Mutation Leads to Motor Neuron Abnormalities

**Figure 3:**
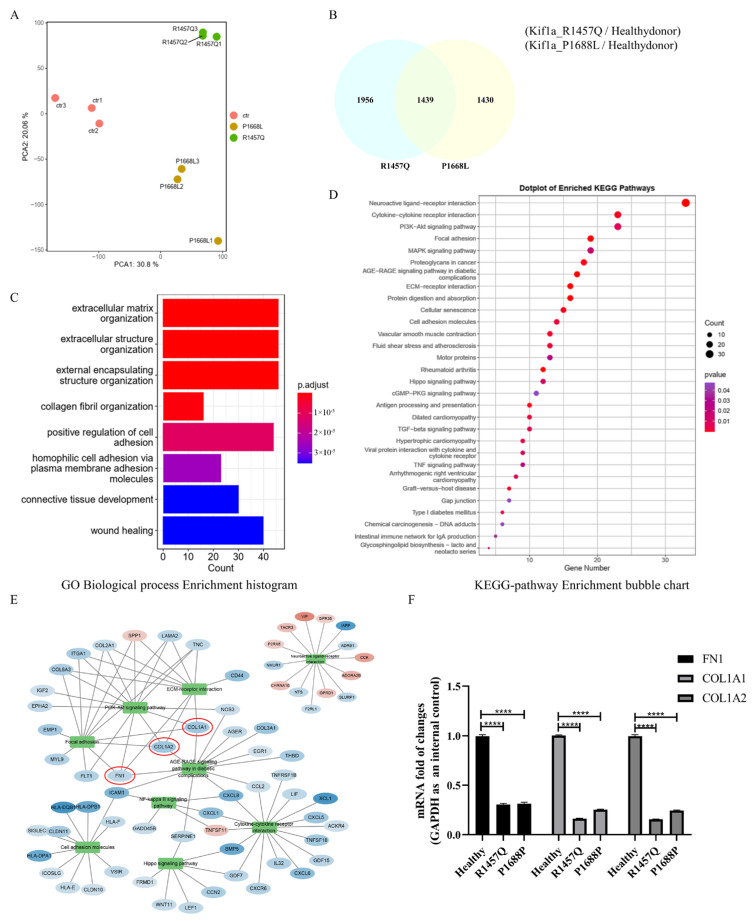
RNAseq analysis of the motor neurons from *KIF1A* mutant and healthy donor control. (**A**) PCA-plot between three sample groups. (**B**) VENN diagram of common differentially expressed genes between the R1457Q/healthy donor and P1688L/healthy donor groups. (**C**) a GO analysis of quantitative RNAseq data of the mature motor neurons from *KIF1A* mutant and healthy donor control. (**D**) KEGG pathway analysis of common differentially expressed genes. (**E**) Building the Gene-Act-Network of the KEGG database. Blue color indicates decreased expression levels in *KIF1A* mutant compared with healthy donor control, while red color indicates increased levels. The depth of the color represents the extent of changes, with log2FC > 0.6 or <−0.6 and FDR < 0.05. (**F**) Validation of RNA-seq data by qRT-PCR of cell adhesion-associated genes. **** *p* < 0.0001. (Student’s *t*-test was used for comparison).

To elucidate the pathogenesis associated with the *KIF1A* mutation, mature motor neurons were collected after 25 days of culture for RNA-seq analysis. First, we examined the PCA plot of the three sample groups. Figure 3A shows that the gene expression in the KIF1A R1457 and P1688L mutant groups was significantly different from that in the healthy donor control group. Next, we performed differential gene expression analysis. We identified a total of 2869 differentially expressed genes in the R1457Q group and 3395 in the P1688L group, compared to the healthy donor group, with 1439 common differentially expressed genes between the two mutant groups (Figure 3B). To analyze the 1439 common differentially expressed genes, we performed GO and KEGG pathway analyses. The results indicated that the functions of the extracellular matrix and structure, as well as cell adhesion, were significantly disturbed (Figure 3C,D). Other disturbed functions included cytokine-cytokine receptor interaction, neuroactive ligand-receptor interaction, ECM-receptor interaction, and cellular senescence. We also performed Gene-Act-Network and hub-gene analysis, which showed that the expression of genes related to cell adhesion, such as *FN1*, *COL1A1*, and *COL1A2*, was significantly downregulated. The qRT-PCR results confirmed these findings (Figure 3E,F). KIF1A is a protein that belongs to the kinesin motor superfamily and plays a crucial role in axonal transport, specifically in the movement of synaptic vesicle precursors and other cargos along microtubules in neurons. Disruption of the transport machinery leads to neuron morphological abnormalities and death [16]. Mutations in the *KIF1A* gene may cause abnormalities in cargo transport, leading to the occurrence of neurological diseases.

### 3.4. KIF1A Mutations Result in Its Accumulation in the Proximal Region of the Axon

**Figure 4:**
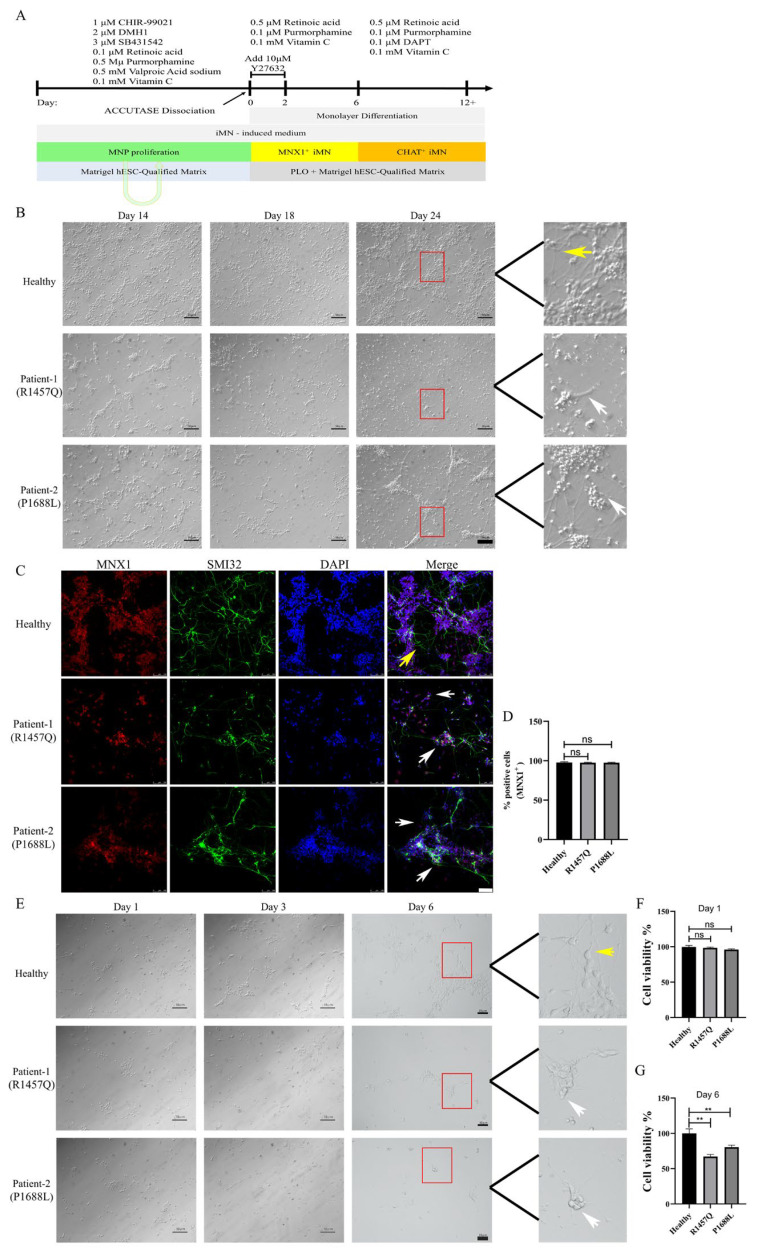
Differentiation of iPSCs to single spinal motor neurons. (**A**) Schematic of the protocol for single motor neuron differentiation. Abbreviations: MNP, motor neuron progenitor; iMN, induced motor neuron; PLO: Poly-L-ornithine. (**B**) Bright-field images showing the differentiation of MNPs into motor neurons from a healthy donor and an ALS patient. Scale bar: 50 μm. (**C**) Immunofluorescence for early motor neuron markers MNX1 and SMI32. Scale bar: 100 μm. (**D**) Quantification of MNX1, n = 5. (**E**) Bright-field images show the differentiation of motor neuron progenitors into single motor neurons. Scale bar: 50 μm. (**F**) Cell viability was detected by CCK-8 assay in motor neuron progenitors differentiation after 1 day. (**G**) Cell viability was detected by CCK-8 assay in motor neuron progenitors differentiation after 6 day. The experiments were performed in triplicate, with values presented as mean ± SD. Normal neurofilament morphology: yellow arrow heads; Abnormal neurofilament morphology: white arrow heads. ** *p* < 0.01, ns, not significant. (Student’s *t*-test was used for comparison).

The above RNA-seq analysis results indicate that the mutation in *KIF1A* leads to reduced neuronal adhesion. We hypothesize that mutations in the C-terminus of KIF1A disrupt cargo transport, resulting in abnormalities in neuronal morphology. However, traditional protocols for differentiating iPSCs into motor neurons using small molecules result in neuronal growth in clusters, making it difficult to quantify and observe neuronal death and pathological changes [17,18,19]. To address this, we adapted the protocol to generate single motor neurons (Figure 4A). The results demonstrate that with the modified motor neuron differentiation protocol, iPSCs from healthy donors can normally differentiate into motor neurons (Figure 4B). Similarly, iPSCs from ALS patients can differentiate into motor neurons with no significant difference in efficiency compared to those from healthy donors. Motor neurons from both healthy donors and ALS patients showed no difference in the ratios of MNX1+ positive neurons (Figure 4C,D). However, during single-cell differentiation of motor neurons, we observed that the density of neurons derived from ALS patients decreased compared to those from healthy donors during their differentiation from neural progenitor cells into motor neurons. (Figure 4B, day 18 and day 24), and the neurofilament morphology was noticeably abnormal (as shown by white arrow heads). The axons of mutant KIF1A MNs exhibited damaged morphologies, such as fragile axons (see SMI32, as shown by white arrow heads) (Figure 4B,C). Consequently, we used the newly adapted protocol to induce iPSC differentiation into individual motor neurons and cultured them in 96-well plates, using CCK-8 assays to evaluate cell viability. The results showed that during the differentiation process from neuronal progenitors to motor neurons, the viability of neurons from ALS patients was significantly reduced, and abnormalities in neuronal morphology were observed (Figure 4E–G).

**Figure 5:**
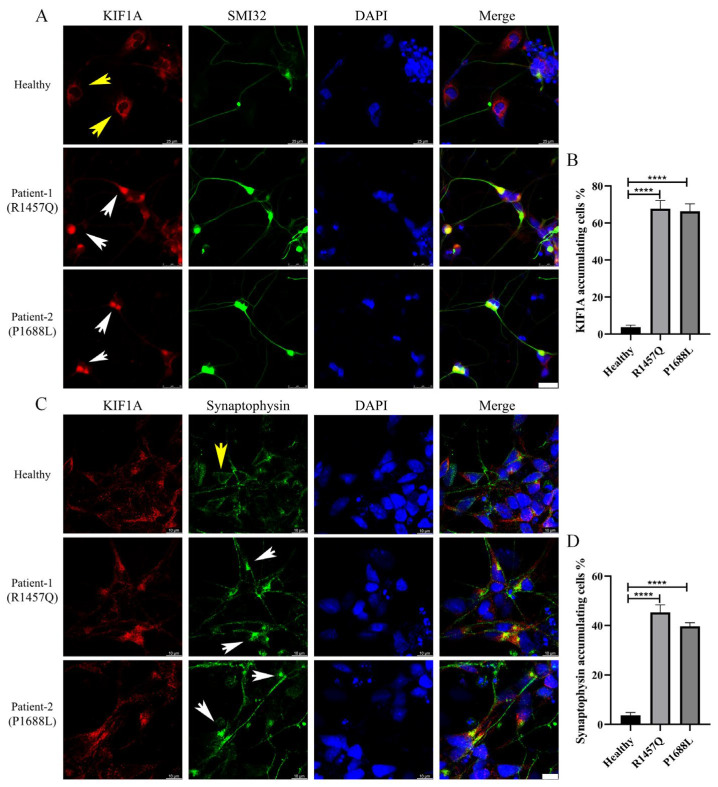
Accumulation of mutant KIF1A in the proximal axon. (**A**) Representative confocal immunofluorescence images of KIF1A (red) in motor neurons, white arrows indicate KIF1A accumulation in the proximal axon. Scale bar: 25 μm. (**B**) Quantify the accumulation of mutant KIF1A in the proximal axon, n = 5. (**C**) Colocalization of synaptophysin with KIF1A in motor neurons and white arrows indicates synaptophysin accumulation in the proximal axon. (**D**) Quantify the accumulation of mutant synaptophysin in the proximal axon, n = 5. Scale bar: 10 μm. Normal distribution of KIF1A or synaptophysin in motor neurons: yellow arrow heads. **** *p* < 0.0001, ns, not significant. (Student’s *t*-test was used for comparison).

In ALS, continual neuronal death is a hallmark, driving progressive motor neuron degeneration and neurological decline [1]. Our established iPSC-MN model effectively recapitulates this disease phenotype. As the ALS-associated KIF1A mutant identified in this study is located at the C-terminal CC4-PH region, which is known to bind cargo [6], we investigated the distribution of KIF1A in motor neurons and the abnormalities in cargo transport. Through immunofluorescence, we found that wild-type KIF1A exhibits a punctate and evenly distributed pattern in the soma and axons of motor neurons (as shown by yellow arrow heads), while the mutant KIF1A accumulates in the proximal region of the axon (as shown by white arrow heads) (Figure 5D,E). Next, we assessed the distribution of synaptophysin in neurons. As one of the cargoes transported by KIF1A and a marker of synaptic vesicles, synaptophysin is crucial for the growth, development, and survival of neurons [20]. The results showed that *KIF1A* mutations not only lead to the accumulation of KIF1A in the proximal region of the axon but also prevent the transport of synaptophysin into the axon, resulting in its accumulation in the proximal axon (Figure 5F,G). This disruption may be a critical underlying mechanism for the observed reduction in neuronal adhesion, morphological abnormalities, and neuronal death associated with *KIF1A* mutations.

### 3.5. KIF1A Mutations Induce Accumulation of Lysosomal Proteins in the Proximal Region of Axons and Autophagy Dysfunction

**Figure 6:**
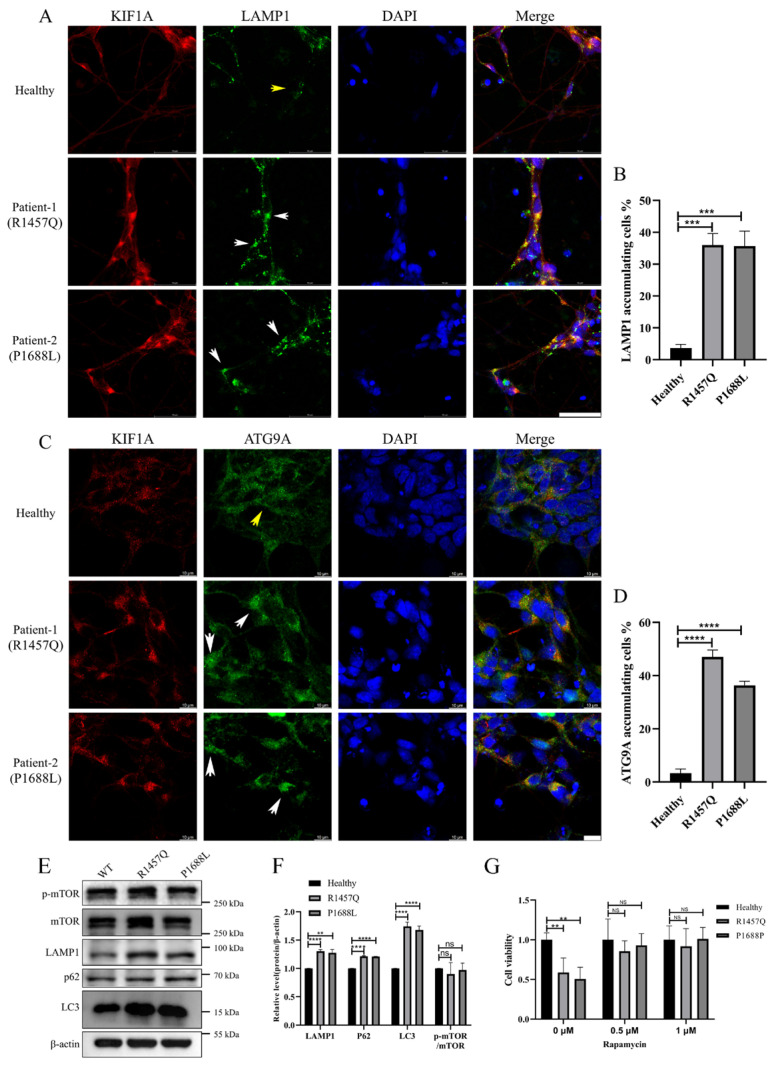
KIF1A mutations induce accumulation of LAMP1 and ATG9A in the proximal region of axons and autophagy dysfunction. (**A**) Colocalization of LAMP1 with KIF1A in motor neurons and white arrows indicates synaptophysin accumulation in the proximal axon. Scale bar: 50 μm. (**B**) Quantifies the accumulation of mutant LAMP1 in the proximal axon, n = 5. (**C**) Colocalization of ATG9A with KIF1A in motor neurons and white arrows indicates synaptophysin accumulation in the proximal axon. Scale bar: 10 μm. (**D**) Quantifies the accumulation of mutant LAMP1 in the proximal axon, n = 5. (**E**) Western blot analysis of autophagy-associated proteins. (**F**) Quantification of (**D**). (**G**) The autophagy activator rapamycin can prevent neuronal death. **** *p* < 0.0001, *** *p* < 0.001, ** *p* < 0.01, ns, not significant. (Student’s *t*-test was used for comparison).

Autophagy plays a critical role in ALS by mediating the clearance of damaged proteins and organelles, potentially mitigating neuronal degeneration and death. In single motor neurons, we observed KIF1A mutant accumulation in the proximal axon. Research indicates that KIF1A is involved in the transport of lysosomes [21,22]. Therefore, we assessed the distribution of the KIF1A transport cargo proteins LAMP1 and ATG9A in neurons, which are lysosome-related proteins. The results showed that KIF1A mutations lead to the abnormal accumulation of LAMP1(Figure 6A,B) and ATG9A (Figure 6C,D) in the proximal axon. Western blot results indicate that KIF1A mutations, while not affecting mTOR activity, lead to abnormal accumulation of LMPA1, P62, and LC3, causing autophagy dysfunction (Figure 6E,F). The abnormal accumulation of KIF1A in the proximal region of the axon may lead to impaired cargo transport, resulting in abnormal neuronal morphology, reduced adhesion, and dysfunction of autophagy, which may be key causes of neuronal death. Interestingly, when we used the autophagy activator rapamycin during motor neuron differentiation, it was able to prevent neuronal death (Figure 6G). The activation of autophagy may play an important role in maintaining neuronal morphology and survival.

## 4. Discussion

In our previous research, we defined a new KIF1A-associated phenotype of ALS and found that mutations in the C-terminal cargo-binding region of KIF1A may lead to the onset of ALS [5]. However, the mechanism is not completely clear, and further research into its molecular mechanism is needed. Here, we established ALS motor neuron models carrying *KIF1A* mutations using iPSC technology, which faithfully recapitulated specific aspects of the disease. Through this model, we found some of the molecular mechanisms by which *KIF1A* mutations contribute to ALS.

In neurons, the transport of cargo into the correct compartments is especially im-portant for many cellular functions. Disruption of the transport machinery leads to neuronal dysfunction and is closely associated with the occurrence of neurological diseases [23,24,25]. For example, Gabrych et al. found that mutations in the molecular motor KIF1A can cause hereditary spastic paraplegias [16]. *KIF1A* encodes a kinesin-3 molecular motor that drives transport to both axons and dendrites. Previously, dense core vesicles (DCVs), lysosomes and synaptic vesicle precursors (SVPs) have been described as cargo for KIF1A [20,21,26,27]. Hummel et al. demonstrated that the CC4 and PH regions are essential for the interaction between KIF1A and its cargo. The absence of these regions leads to abnormal KIF1A distribution, preventing it from localizing to the distal axon [6]. As the ALS-associated KIF1A mutant identified in this study is located at the C-terminal CC4-PH region, which binds cargo, we primarily focus on the distribution of KIF1A in motor neurons and the abnormalities in cargo transport. Through the iPSC-iMN model, we found that KIF1A pathogenic mutations cause abnormal aggregation in the proximal axon, with its transport cargo, synaptophysin, LAMP1 and ATG9A, also accumulating there.

As a transport cargo of KIF1A, LAMP1 is a protein located on the lysosomal membrane and plays a crucial role in lysosomal maturation and the fusion of autophagosomes with lysosomes during autophagy [21,28]. Autophagy regulates axon morphogenesis and function in neurons and is essential for neuronal survival. Studies have shown that autophagy dysfunction leads to axonal degeneration and neuronal cell death, and that autophagy activators can safely and effectively treat ALS [22,29]. In this study, we found that KIF1A mutations lead to the abnormal accumulation of LAMP1, p62, and LC3, causing autophagy dysfunction and neuronal death. Interestingly, when we supplemented rapamycin during motor neuron differentiation, we found that it effectively prevented motor neuron death. This finding suggests that activating autophagy can partially ameliorate the dysfunction resulting from aberrant cargo transport caused by KIF1A mutations.

Abnormal neuronal cell adhesion, linked to alterations in cell adhesion molecules, plays a critical role in neurodegenerative diseases by disrupting axon guidance and synapse formation [30,31,32]. Recent research demonstrates that mutations in *PCDHA9* (protocadherin alpha 9) result in the instability and dysfunction of the protein it encodes, which significantly impair neuronal transport functions and cell adhesion mechanisms. These disturbances are critically linked to the pathogenesis of amyotrophic lateral sclerosis [33]. KIF1A transports various substances crucial for neuronal development and morphological maintenance, such as BDNF and synaptophysin [21,27]. Mutations in the cargo-binding region of KIF1A can lead to its abnormal aggregation at the proximal axon, potentially resulting in cargo failing to reach specific synaptic locations, thus affecting neuronal morphology and survival. Through RNAseq analysis, we observed that mutations in *KIF1A* significantly disrupt motor neuron functions related to the extracellular matrix, structure, and cell adhesion. We hypothesize that the autophagy dysfunction caused by the abnormal aggregation of LAMP1 due to KIF1A mutations may also be related to decreased neuronal cell adhesion and death, as autophagy is crucial for neuronal survival and the integrity of synaptic structures.

Currently, the iPSC-iMN models retain the patient’s full genetic information, increasing opportunities for disease mechanism research and drug screening, and offering theoretical insights for ALS treatment. In this study, we used the episomal vector method for iPSC reprogramming [34,35]. During the reprogramming of peripheral blood T cells from volunteers, we discovered that the success rate of reprogramming is closely related to the vitality of the T cells. We found that activating T cells with CD3/CD28 and IL-2 after introducing the reprogramming factors (Oct4, Sox2, Klf4, and L-Myc) into the cells significantly increased the success rate of generating iPSCs. This is a very interesting finding. Furthermore, we have modified traditional iPSC-MN protocols to generate individual motor neurons, which better replicate the pathological phenotypes of ALS patient-specific neurons carrying *KIF1A* pathogenic mutations [15,17,18,19]. We found that when iPSCs are induced to differentiate into motor neuron progenitor cells, cell clusters can be dissociated into single cells using ACCUTASE. Temporarily adding Y27632 allows neural progenitor cells to efficiently differentiate into motor neurons in culture dishes coated with Poly-L-ornithine (PLO) and Matrigel. Through this adapted model, we can more clearly study the molecular mechanisms of ALS pathogenic mutations.

In summary, we have generated an iPSC-MN disease model carrying *KIF1A* mutations, enabling the first investigation into the pathogenic mechanism of ALS resulting from heterozygous mutations in endogenous *KIF1A*. We found that KIF1A mutations (p.Arg1457Gln and p.Pro1688Leu) lead to its accumulation in the proximal axon and cause its transport cargo to accumulate there, resulting in significantly disturbed cell-cell adhesion and autophagy dysfunction. Using the autophagy activator rapamycin can prevent neuronal death. Although we have not explained why KIF1A p.Arg1457Gln and p.Pro1688Leu mutations lead to their abnormal accumulation at the proximal end of axons, we provide new evidence for the molecular mechanisms by which *KIF1A* may cause the onset of ALS.

## Data Availability

The data used in this study are available from the corresponding author upon reasonable request.

## Supplementary Materials

The following supporting information can be downloaded at: https://www.mdpi.com/article/10.3390/biomedicines12081693/s1, Figure S1. Differentiation of iPSCs into motor neuron. Related to Figure 2.
