# Supplementary material for: KIF1A, R1457Q, and P1688L Mutations Induce Protein Abnormal Aggregation and Autophagy Impairment in iPSC-Derived Motor Neurons
Source: Biomedicines. 2024 Jul 30;12(8):1693. doi: 10.3390/biomedicines12081693 (PMC11351720; doi:10.3390/biomedicines12081693)
Supplement: Supplementary file 1 [file biomedicines-12-01693-s001.zip › biomedicines-3094694-supplementary.pdf]

Supplemental Information

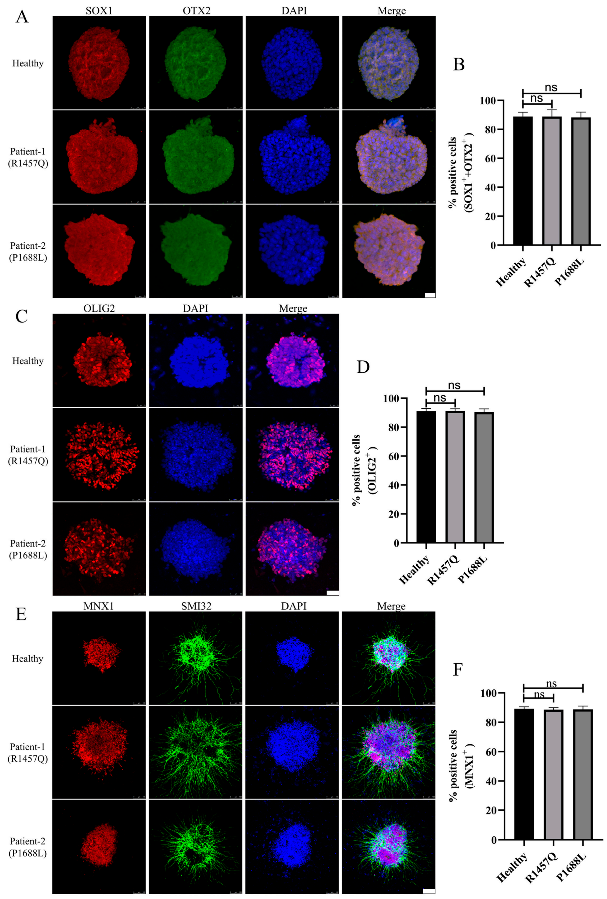

Figure S1. Differentiation of iPSCs into motor neuron. Related to Figure 2.

(A) Immunofluorescence for neuroepithelial progenitor markers SOX1 and OTX2 on day 6. Nuclei were stained with DAPI. Scale bar: 50 μm. (B) Through the quantification of (A), n = 5. (C) Immunofluorescence for motor neuron progenitor markers OLIG2 on day 12. Scale bar: 50 μm. (D) Through the quantification of (C), n = 5. (E) Immunofluorescence for early motor neuron markers MNX1 and SMI32 on day 18. SMI32 marks axons by targeting non-phosphorylated neurofilaments. Scale bar: 100 μm. (F) Through the quantification of (E), n = 5. Data are shown as mean ± SD (ns, not significant; Student's t-test was used for comparison).
